# Effects of eight weeks of aerobic interval training and of isoinertial resistance training on risk factors of cardiometabolic diseases and exercise capacity in healthy elderly subjects

**DOI:** 10.18632/oncotarget.4031

**Published:** 2015-05-28

**Authors:** Paolo Bruseghini, Elisa Calabria, Enrico Tam, Chiara Milanese, Eugenio Oliboni, Andrea Pezzato, Silvia Pogliaghi, Gian Luca Salvagno, Federico Schena, Roberto Pozzi Mucelli, Carlo Capelli

**Affiliations:** ^1^ School of Sport and Exercise Sciences, Department of Neurological and Movement Sciences, University of Verona, Verona, Italy; ^2^ Section of Clinical Chemistry, School of Medicine Policlinico “GB Rossi”, Department of Life and Reproduction Sciences, School of Medicine, University of Verona, Verona, Italy; ^3^ Institute of Radiology, School of Medicine, Policlinico “GB Rossi”, Department of Pathology and Diagnostics, School of Medicine, University of Verona, Verona, Italy; ^4^ Norwegian School of Sport Sciences, Department of Physical Performances, Sognsveien, Oslo, Norway

**Keywords:** Gerotarget, high intensity interval training, isoinertial resistance training, aging, metabolic syndrome, cardiovascular fitness

## Abstract

We investigated the effect of 8 weeks of high intensity interval training (HIT) and isoinertial resistance training (IRT) on cardiovascular fitness, muscle mass-strength and risk factors of metabolic syndrome in 12 healthy older adults (68 yy ± 4). HIT consisted in 7 two-minute repetitions at 80%–90% of ${\dot{\text{V}}\text{O}}_{2\max}$, 3 times/w. After 4 months of recovery, subjects were treated with IRT, which included 4 sets of 7 maximal, bilateral knee extensions/flexions 3 times/w on a leg-press flywheel ergometer. HIT elicited significant: i) modifications of selected anthropometrical features; ii) improvements of cardiovascular fitness and; iii) decrease of systolic pressure. HIT and IRT induced hypertrophy of the quadriceps muscle, which, however, was paralleled by significant increases in strength only after IRT. Neither HIT nor IRT induced relevant changes in blood lipid profile, with the exception of a decrease of LDL and CHO after IRT. Physiological parameters related with aerobic fitness and selected body composition values predicting cardiovascular risk remained stable during detraining and, after IRT, they were complemented by substantial increase of muscle strength, leading to further improvements of quality of life of the subjects.

## INTRODUCTION

Current life styles and health policies in Western countries have led to substantial increase of lifespan, but also to a marked decrease of physical activity. This has fostered the progressive diffusion, already among middle-aged adults and even more in elderly subjects, of metabolic diseases that expose these patients to increased cardiovascular morbidity.

Metabolic syndrome affects from 10% to 34% [1, 2] of the populations in Western countries. Its main risk factors are hypertension at rest, insulin resistance with elevated fasting plasma glucose, dyslipidaemia, and visceral adiposity [3]. It is often associated with impaired endothelial functions [4], prothrombotic state [5] and a low level of physical fitness [6]. It exposes the patients to a two-to-fourfold increase in the risk of cardiovascular diseases [7] and cardiovascular mortality [8], placing a huge economic burden on public health services. For instance, in Germany alone, the projected burden will be euro 24.447 million by 2020 [2].

Some of correlates of the metabolic syndrome can be effectively treated with pharmacological interventions; however, modifiable lifestyle changes, including healthier diet and increased physical activity, are the first-line approaches to primary and secondary prevention. Among lifestyle interventions, exercise training may decrease the cardiometabolic risk independently of nutritional and medical therapies [9] leading also to the increase of maximal oxygen uptake (${\dot{\text{V}}\text{O}}_{2\max}$) [10]. This is particularly important for improving and maintaining health, since low ${\dot{\text{V}}\text{O}}_{2\max}$ *per se* is an independent and strong predictor of cardiovascular mortality [11].

Furthermore, exercise training, in addition to contrast the decline in maximal aerobic power and exercise capacity [12], has further positive effects on the health of older adults, as it provides an effective countermeasure against other ominous correlates of ageing, for instance the progressive increase loss of lean body mass [13].

The loss of muscular mass is of particular importance because daily life independence is strongly and negatively affected by the loss of muscle mass (sarcopenia) and by the morphological and biochemical modifications of the skeletal muscle. It is estimated that, on the average, about 5% of muscle mass is lost after the age of 40 [14]. In particular, there seems to occur an age-related loss of muscle fibres [15] with a selective loss of Type-II fibres and a shift to a larger proportion of slow, oxidative Type-I fibres independently from any long lasting endurance-training program. The functional consequences of sarcopenia are obvious and several studies have highlighted the negative impact of muscle loss on strength [16, 17] that is evident even when corrected for muscle loss. This suggests that also the quality and efficiency of the ageing muscles are decreased, as shown by the decline in force per unit of muscle cross sectional area [18, 19].

In addition to the progressive loss of muscle strength, sarcopenia is accompanied also by evident metabolic consequences. The progressive decrease of lean body mass is mirrored by the decay of resting metabolic rate [20] and implies a decrease in daily physical activity and in total expenditure per day [14, 20, 21]. Both these factors predispose elderly to accumulate visceral [22] and total body fact and to be prone to poor insulin sensibility and to increased post-prandial hyperglycaemia. Finally, the loss of muscle mass has also more subtle, yet very important, connections with the decay of ${\dot{\text{V}}\text{O}}_{2\max}$ observed with ageing [14]. According to a multifactorial mode of the factors limiting ${\dot{\text{V}}\text{O}}_{2\max}$, maximal cardiovascular oxygen delivery (${\dot{\text{Q}}}_{\text{a}}{\text{O}}_{2\max}$) provides about 70% of the fractional limitation of ${\dot{\text{V}}\text{O}}_{2\max}$ in normoxic condition [23]. The factors influencing peripheral gas exchanges, i.e. O_2_ diffusion and utilization accounts for the remaining 30% of the total fractional limitation. Therefore, we may hypothesise that both the loss of lean muscle mass and the peripheral modifications that impair peripheral gas exchanges may concur, together with the decay of ${\dot{\text{Q}}}_{\text{a}}{\text{O}}_{2\max}$, to the progressive drop of ${\dot{\text{V}}\text{O}}_{2\max}$ observed in ageing. This hypothesis is somehow strengthened by the observation that the slow component of the decay of ${\dot{\text{V}}\text{O}}_{2\max}$ observed after two months of head down tilt bed rest in volunteers undergoing prolonged inactivity mirrors the parallel decrease in muscle mass of the lower limbs [24].

To summarize, we may hypothesize that by contrasting the loss muscle mass with appropriate resistance training we may maintain and / or potentiate the beneficial adaptations induced by aerobic training on the main risk factors of the metabolic syndrome even in healthy, elderly subjects and, at the same time, contrast the ominous consequences of sarcopenia.

Current guidelines recommend at least 150 minutes per week of moderate intensity exercise (50% to 70% maximal heart rate, HR_max_) or 75 minutes per week of vigorous intensity exercise (70% to 80% HR_max_) for adults [25]. In the general population, however, these targets are difficult to achieve. For instance, only about 65% of adults in the United States meet these target recommendations [25]. The main barrier to reaching recommended goals seems to be time constraints, a common problem in many Western countries because of the gradual changes in labour participation [26]. Experimental evidence suggests that high-intensity interval training (HIT) may be an efficient alternative to continuous moderate aerobic exercise (CME) and suitable for exercise-based therapies in large groups of older adults and in patients with metabolic syndrome [27, 28, 29]. In brief, HIT may be a more time-efficient approach than CME to achieve specific health benefits in large groups of older adults. Nevertheless, the efficacy of HIT in healthy older adults has been seldom evaluated. For instance, one study investigated the effects of HIT on selected cardiovascular risk factors [30]; the second studied the effects of interval training at the ventilatory threshold on cardiorespiratory responses [31].

Resistance training is highly effective when concentric and eccentric contractions are repeatedly applied [32]. Isoinertial ergometer [33], by applying the flywheel principle, is able to generate resistance force during both the lengthening and shortening actions of the contraction. This is achieved by storing energy input in the shortening phase of a movement thanks to a lightweight plastic flywheel's rotation; this force is then used to provide resistance during the lengthening action of each movement.

On the basis of the exposed premises, in the present small sample, proof-of-concept investigation, twelve moderately active older adults were exposed to eight weeks of HIT training with seven two-minute repetitions at 85%–95% of ${\dot{\text{V}}\text{O}}_{2\max}$, three times a week. After four months of recovery, the same volunteers underwent eight weeks of isoinertial resistance (IRT) training. The study was aimed at documenting to what extent HIT and IRT may be effective for reducing selected risks of with metabolic syndrome and cardiometabolic diseases, modifying anthropometrical variables, improving cardiopulmonary fitness and exercise resistance and influencing muscle mass and performances in this particular group of subjects. Moreover, we aimed to confirm whether the main determinants of aerobic fitness are less negatively affected in these subjects during detraining if they maintain their normal levels of daily activities.

## RESULTS

### Body composition and muscle structure

The results concerning body composition and muscle structure are presented in Table 1. ANOVA analysis showed that per cent body fat (BF, %) (*P* = 0.00002, = 0.392) and abdominal fat (AF, %) were significantly different (*P* = 0.0165, = 0.156). In particular, BF and AF were larger before HIT than in all the other conditions (Table 1). WC decreased after HIT. Waist to height ratio (W/Height) at Post HIT, Pre IRT and Post IRT were significantly smaller than at Pre HIT. CSA turned out to be significantly different in the four conditions (*P* = 0.00063, = 0.281): CSA at Post HIT and at Post IRT was significantly larger than at Pre HIT and Pre IRT, respectively. The value after resistance training was also significantly larger than in Pre HIT. Quadriceps muscle volume (Vol, cm^3^) showed a similar pattern, as it was significantly different in the four conditions (*P* = 0.00050, = 0.289): Vol at Post HIT and at Post IRT was significantly larger than at Pre HIT and Pre IRT, respectively; finally, it was significantly larger after resistance training than in Pre HIT.

**Table 1:** Subjects' value before (Pre HIT) and after (Post HIT) High Intensity Interval Training and before (Pre IRT) and after (Post IRT) isoinertial resistance training (mean, SD) *P*-value (*P*), partial omega squared of single contrast (${\hat{\omega }}_{\varphi }^{2}$)

	Condition			
	Pre HIT	Post HIT	Pre IRT	Post IRT
BW (kg)	77.8 ± 10.4	76.8 ± 9.9	76.3 ± 9.3	76.7 ± 8.9
BMI (kg m^−2^)	26.5 ± 2.8	26.2 ± 2.6	25.9 ± 2.4	26.0 ± 2.5
BF (%)	26.2 ± 4.3	25.2^*^ ± 6.2	24.6^*^ ± 5.7	24.6^*^ ± 5.5
*P*		0.0029	0.0002	0.0010
${\hat{\omega }}_{\varphi }^{2}$		0.309	0.493	0.389
AF (%)	32.5 ± 9.4	31.1^*^ ± 9.7	30.4^*^ ± 8.3	30.5^*^ ± 8.4
*P*		0.0238	0.0137	0.2203
${\hat{\omega }}_{\varphi }^{2}$		0.142	0.1860	0.148
LBM (kg)	56.9 ± 6.2	57.1 ± 5.29	57.3 ± 5.9	57.6^†^ ± 5.8
*P*				0.0431
${\hat{\omega }}_{\varphi }^{2}$				0.0959
WC (cm)	95.2 ± 9.7	93.9^*^ ± 9.2	93.3 ± 8.0	93.5 ± 8.1
*P*		0.0028		
${\hat{\omega }}_{\varphi }^{2}$		0.235		
W/Hip	0.92 ± 0.07	0.92 ± 0.06	0.84 ± 0.24	0.92 ± 0.06
W/Height	0.56 ± 0.05	0.55^*^ ± 0.05	0.54^*^ ± 0.04	0.54^*^ ± 0.03
*P*		0.0158	0.0074	0.0199
${\hat{\omega }}_{\varphi }^{2}$		0.174	0.234	0.156
CSA (cm^2^)	60.3 ± 10.6	62.9^*^ ± 10.5	59.5 ± 9.3	62.0^†^,^*^ ± 9.3
*P*		0.0158		0.0199
${\hat{\omega }}_{\varphi }^{2}$		0.174		0.156
Vol (cm^3^)	820 ± 198	865^*^ ± 199	812 ± 184	852^†^,^*^ ± 188
*P*		0.0111		0.0089/0.0036
${\hat{\omega }}_{\varphi }^{2}$		0.218		0.100/0.283

* significantly different from the value at Pre HIT

† significantly different from the value at Pre IRT.

BW: body weight; BMI: Body Mass Index; Fat: per cent body fat; AF: per cent android fat; LBM: lean body mass; WC: waist circumference; W/Hip: waist to hip ratio; W/height: waist to height ratio; CSA: Muscular Cross Sectional Area; Vol: Muscular volume.

### Maximal test

In Table 2, the results of the maximal exercise test, including the quantification of the gas exchange thresholds, are presented. Maximal mechanical power attained during the maximal test (W_max_, W) was significantly different in the evaluated conditions (*P* = 0.00001 = 0.413). In particular, it was significantly smaller at Pre HIT than in the other three occasions. Absolute ${\dot{\text{V}}\text{O}}_{2\max}$ (L min^−1^) was significantly larger after HIT than before. When normalized by the measured body mass (mL min^−1^kg^−1^), it turned out to be significantly smaller at Pre HIT than in the other three occasions. Maximal O_2_ pulse (PO_max_, mL pulse^−1^) after HIT and before IRT was significantly larger than before aerobic high intensity training. ${\dot{\text{V}}\text{O}}_{2}$ at GET (mL min^−1^kg^−1^) turned out to be significantly larger at Pre (*P* = 0.01119, = 0.196) and Post IRT (*P* = 0.04228, = 0.098) than at the beginning of the study. As for ${\dot{\text{V}}\text{O}}_{2}$ at RCP (mL min^−1^kg^−1^), it turned out to be significantly different in the various occasions (*P* = 0.00009, = 0.346). In particular, it was significantly smaller at Pre HIT than at Post HIT, Pre IRT and Post IRT. Finally, also the metabolic power at RCP expressed as per cent of ${\dot{\text{V}}\text{O}}_{2\max}$ was significantly different in the four experimental sessions (*P* = 0.00037, = 0.300), being larger at Post HIT, Pre IRT and Post IRT than at Pre HIT.

**Table 2:** Subjects' value before (Pre HIT) and after (Post HIT) High Intensity Interval Training and before (Pre IRT) and after (Post IRT) isoinertial resistance training (mean, SD) *P*-value (*P*), partial omega squared of single contrast (${\hat{\omega }}_{\varphi }^{2}$)

	Condition			
	Pre HIT	Post HIT	Pre IRT	Post IRT
W_max_ (Watt)	199 ± 37	219^*^ ± 33	216^*^ ± 35	218^*^ ± 38
*P*		0.0002	0.0005	0.0032
${\hat{\omega }}_{\varphi }^{2}$		0.502	0.445	0.299
${\dot{\text{V}}\text{O}}_{2\max}$ (L min^−1^)	2.34 ± 0.35	2.48^*^ ± 0.38	2.43 ± 0.43	2.44 ± 0.42
*P*		0.0125		
${\hat{\omega }}_{\varphi }^{2}$		0.192		
${\dot{\text{V}}\text{O}}_{2\max}$ (mL min^−1^kg^−1^)	29.9 ± 4.3	32.6^*^ ± 6.0	32.0^*^ ± 5.4	32.1^*^ ± 6.1
*P*		0.0032	0.0088	0.0341
${\hat{\omega }}_{\varphi }^{2}$		0.300	0.220	0.114
PO_max_ (mL pulse^−1^)	15.31 ± 2.46	16.45^*^ ± 2.51	16.23^*^ ± 2.92	16.20 ± 2.96
*P*		0.0126	0.0143	
${\hat{\omega }}_{\varphi }^{2}$		0.192	0.182	
GET (mL min^−1^kg^−1^)	17.6 ± 3.8	18.4 ± 4.2	19.9^*^ ± 5.0	19.5^*^ ± 5.8
*P*			0.0119	0.0423
${\hat{\omega }}_{\varphi }^{2}$			0.196	0.098
GET_%_ (%${\dot{\text{V}}\text{O}}_{2\max}$)	58.8 ± 8.4	55.9 ± 5.0	61.9 ± 8.04	60.1 ± 8.8
RCP (mL min^−1^kg^−1^)	22.8 ± 4.3	27.1^*^ ± 5.1	27.7^*^ ± 5.2	27.7^*^ ± 6.3
*P*		0.0002	0.0006	0.0005
${\hat{\omega }}_{\varphi }^{2}$		0.502	0.422	0.435
RCP_%_ (%${\dot{\text{V}}\text{O}}_{2\max}$)	76.4 ± 8.3	82.9^*^ ± 4.9	86.4^*^ ± 5.3	85.6^*^ ± 5.02
*P*		0.0113	0.0026	0.0019
${\hat{\omega }}_{\varphi }^{2}$		0.201	0.315	0.340

* significantly different from the value at Pre HIT

^†^ significantly different from the value at Pre IRT.

W_max_: maximal mechanical power; ${\dot{\text{V}}\text{O}}_{2\max}$: maximal oxygen uptake; PO_max_: maximal oxygen pulse; GET: oxygen uptake at the Gas Exchange Threshold; GET_%_: Gas Exchange Threshold as per cent of ${\dot{\text{V}}\text{O}}_{2\max}$; RCP: oxygen uptake at the Respiratory Compensation Point; RCP_%_: Respiratory Compensation Point as per cent of ${\dot{\text{V}}\text{O}}_{2\max}$.

### Exercise efficiency

Gross efficiency of cycling exercise during submaximal exercise did not change throughout the study and ranged from 0.147 ± 0.011 at Post IRT to 0.150 ± 0.014 at Pre IRT.

### Blood analysis

In Table 3 the average values of blood analysis data obtained in the four tests are shown. GLU was significantly different among the different sessions (*P* = 0.01210, = 0.169). GLU at Pre HIT was significantly larger than at Post HIT and Pre IRT. After strength training, however, GLU turned out to be larger than before training. CHO (mmol L^−1^) was smaller than at the beginning of the study only Post IRT. HDL (mmol L^−1^) was found to be significantly lower only at Pre IRT. LDL (mmol L^−1^) were significantly different (*P* = 0.01893, = 0.149); LDL after IRT training was significantly lower than at Pre HIT, Pre IRT and Post HIT. CRP (mg L^−1^) was significantly smaller after IRT than Post HIT.

**Table 3:** Subjects' value before (Pre HIT) and after (Post HIT) High Intensity Interval Training and before (Pre IRT) and after (Post IRT) isoinertial resistance training (mean, SD) *P*-value (*P*), partial omega squared of single contrast (${\hat{\omega }}_{\varphi }^{2}$)

	Condition			
	Pre HIT	Post HIT	Pre IRT	Post IRT
GLU (mmol L^−1^)	5.03 ± 0.55	4.68^*^ ± 0.69	4.61^*^ ± 0.75	4.85^†^ ± 0.76
*P*		0.0004	0.0030	0.0440
${\hat{\omega }}_{\varphi }^{2}$		0.282	0.304	0.095
CHO (mmol L^−1^)	5.62 ± 1.26	5.38 ± 0.82	5.28 ± 1.13	5.22^*^ ± 0.84
*P*				0.0414
${\hat{\omega }}_{\varphi }^{2}$				0.099
HDL (mmol L^−1^)	1.52 ± 0.36	1.56 ± 0.33	1.46^*^ ± 0.29	1.52 ± 0.35
*P*			0.0310	
${\hat{\omega }}_{\varphi }^{2}$			0.121	
LDL (mmol L^−1^)	3.66 ± 0.81	3.77 ± 0.35	3.77 ± 0.80	3.29^†^, ^¶^, ^*^ ± 0.62
*P*				0.0005/0.0025/0.0145
${\hat{\omega }}_{\varphi }^{2}$				0.437/0.318/0.176
CHO/HDL	3.75 ± 0.68	3.58 ± 0.87	3.72 ± 0.88	3.62 ± 0.99
TRY (mmol L^−1^)	1.23 ± 0.38	1.20 ± 0.41	1.18 ± 0.40	1.19 ± 0.45
CRP (mg L^−1^)	3.3 ± 0.9	3.0 ± 0.3	3.9 ± 3.5	3.8^¶^ ± 1.38
*P*				0.0329
${\hat{\omega }}_{\varphi }^{2}$				0.117
Hb (mg dL^−1^)	15.3 ± 0.9	15.4 ± 0.9	15.7^*^ ± 1.0	15.2^†^ ± 1.25
*P*			0.0310	0.0078
${\hat{\omega }}_{\varphi }^{2}$			0.119	0.229

* significantly different from the value at Pre HIT

† significantly different from the value at Pre IRT

¶ significantly different from the value at Post HIT.

GLU: fasting glucose; CHO: total cholesterol; HDL: high-density lipoprotein; LDL: low-density lipoprotein;

CHO/HDL: total cholesterol/HDL ratio; TRY: triglyceride; CRP: c-reactive protein; Hb: hemoglobin.

### Blood pressure

Table 4 presents the average values of arterial blood pressure prevailing in the four experimental sessions. Arterial diastolic pressure (DP, mm Hg) before the resistance training was smaller than at Pre HIT. Systolic pressure (SYS, mm Hg) was significantly different in the four experimental sessions (*P* = 0.00002, = 0.397). In particular, it was significantly larger in the control condition, before the interventions, than Post HIT, Pre IRT and Post IRT. The value measured after the IRT was significantly larger than that prevailing before strength training and after HIT. Mean arterial pressure (MP, mm Hg) showed a pattern similar to DP being significantly different in the four occasions (*P* = 0.00490, = 0.205); in particular, MP was significantly smaller at Post HIT and Pre IRT than at Pre HIT. Conversely, the values at Post IRT were significantly larger than those before strength training and than the ones prevailing after high intensity, interval aerobic training.

**Table 4:** Subjects' value before (Pre HIT) and after (Post HIT) High Intensity Interval Training and before (Pre IRT) and after (Post IRT) isoinertial resistance training (mean, SD) *P*-value (*P*), partial omega squared of single contrast (${\hat{\omega }}_{\varphi }^{2}$)

	Condition			
	Pre HIT	Post HIT	Pre IRT	Post IRT
DP (mm Hg)	85 ± 10	82 ± 5	81^*^ ± 7	84 ± 10
*P*			0.0485	
${\hat{\omega }}_{\varphi }^{2}$			0.087	
SYS (mm Hg)	140 ± 16	128^*^ ± 9	128^*^ ± 11	134^†^,^¶^, ^*^ ± 10
*P*		0.0003	0.0004	0.0207/0.0040/0.0025
${\hat{\omega }}_{\varphi }^{2}$		0.468	0.460	0.153/0.282/0.146
MP (mm Hg)	104 ± 11	97^*^ ± 6	97^*^ ± 8	101^†^,^¶^ ± 9
*P*		0.0112	0.0048	0.0320/0.0251
${\hat{\omega }}_{\varphi }^{2}$		0.202	0.269	0.119/0.138

* significantly different from the value at Pre HIT

† significantly different from the value at Pre IRT

¶ significantly different from the value at Post HIT.

DP: arterial diastolic pressure; SYS: arterial systolic pressure; MP: arterial mean pressure.

### Muscle strength

Mean values of the muscle strength data are presented in Table 5. Maximal concentric isokinetic torque at low (60° s^−1^ C, N m) and at highest (120° s^−1^ C, N m) angular speeds were significantly different (*P* = 0.00010, = 0.342 and *P* = 0.00428, = 0.210, respectively). Specifically, they were significantly larger at Post IRT than in the other three evaluated conditions. Maximal, eccentric isokinetic torque at low (60° s^−1^ E, N m) and at high (120° s^−1^ E, N m) angular speeds were significantly larger than Pre HIT and Pre IRT, respectively. Maximal voluntary isometric torque of leg extensors measured at 60° of flexion (60°_MVC_, N m) was significantly different (*P* = 0.00858, = 0.143). It was larger after IRT than before IRT and after HIT than before aerobic training. Finally, maximal voluntary isometric torque of leg extensors measured at 90° of flexion (60°_MVC_, N m) after IRT was larger, and significantly so, than before IRT and before HIT.

**Table 5:** Subjects' value before (Pre HIT) and after (Post HIT) High Intensity Interval Training and before (Pre IRT) and after (Post IRT) isoinertial resistance training (mean, SD) *P*-value (*P*), partial omega squared of single contrast (${\hat{\omega }}_{\varphi }^{2}$)

	Condition			
	Pre HIT	Post HIT	Pre IRT	Post IRT
60° s^−1^ C (N m)	160 ± 24	163± 22	164 ± 26	179^†^,^¶^,^*^ ± 31
*P*				0.0005/0.0096/0.0004
${\hat{\omega }}_{\varphi }^{2}$				0.441/0.213/0.454
120° s^−1^ C (N m)	130 ± 23	133 ± 24	132 ± 23	139^†^,^¶^,^*^ ± 23
*P*				0.0032/0.0180/0.0049
${\hat{\omega }}_{\varphi }^{2}$				0.300/0.165/0.267
60° s^−1^ E (N m)	238 ± 30	247 ± 24	241 ± 26	255^*^ ± 35
*P*				0.0102
${\hat{\omega }}_{\varphi }^{2}$				0.126
120° s^−1^ E (N m)	230 ± 25	239 ± 26	232 ± 26	245^†^ ± 31
*P*				0.0010
${\hat{\omega }}_{\varphi }^{2}$				0.384
60°_MVC_ (N m)	200 ± 21	215^*^ ± 32	202 ± 23	223^†^,^*^ ± 39
*P*		0.0416		0.0143/0.0169
${\hat{\omega }}_{\varphi }^{2}$		0.099		0.182/0.168
90°_MVC_ (N m )	169 ± 34	165 ± 31	166 ± 38	177^†^,^*^ ± 42
*P*				0.0287/0.0480
${\hat{\omega }}_{\varphi }^{2}$				0.152/0.088

* significantly different from the value at Pre HIT

† significantly different from the value at Pre IRT

¶ significantly different from the value at Post HIT.

60° s^−1^ C: maximal isokinetic concentric torque at 60° s^−1^; 120° s^−1^ C: maximal isokinetic concentric torque at 120° s^−1^;

60° s^−1^ E: maximal isokinetic eccentric torque at 60° s^−1^; 120° s^−1^ E: maximal isokinetic eccentric torque at 120° s^−1^;

60°_MVC_: maximal isometric torque at 60° of knee flexion; 90°_MVC_: maximal isometric torque at 90° of knee flexion.

### Correlations between anthropometrics, muscle volume and physical fitness

The increase of quadriceps muscle Vol (%) measured after IRT was strongly correlated with the consensual per cent change in Lean Body mass (LBM, %) (*r* = 0.72, *P* = 0.008, *n* = 12) and the per cent decrease of AF (*r* = −0.66, *P* = 0.019, *n* = 12) and BF (*r* = −0.60, *P* = 0.039, *n* = 12). Per cent decreases of WC and W/height occurring during HIT were strongly correlated (*r* = 0.95, *P* = 0.0001, *n* = 12).

The drop of GLU (%) induced by HIT was also strongly correlated with the increase (%) of LBM (*r* = −0.52, *P* = 0.086, *n* = 12).

The per cent increase of maximal concentric torque of limb extensors at 60° s^−1^ was strongly related (*r* = 0.804, *P* = 0.002, *n* = 12) with the increase (%) of Vol (*r* = 0.804, *P* = 0.002, *n* = 12) and with that (%) of CSA (*r* = 0.561, *P* = 0.058, *n* = 12); also the increase (%) of maximal eccentric torque at the same angular speed was strongly correlated with Vol (*r* = 0.815, *P* = 0.001, *n* = 12). At the highest angular speed of 120° s^−1^, per cent increase of maximal concentric torque was strongly related with the augmentation (%) of Vol (*r* = 0.872, *P* < 0.001, *n* = 12) and moderately correlated with the increase of CSA (*r* = 0.392, *P* = 0.1, *n* = 12); the increase of maximal eccentric torque (%) at this angular speed was strongly correlated (*r* = 0.557, *P* = 0.060, *n* = 12) only with Vol. During maximal isometric contraction, only the per cent increase of MVC at 60° of knee flexion (60°_MVC_) was moderately correlated with the corresponding increase (%) of Vol (*r* = 0.439, *P* = 0.153, *n* = 12).

## DISCUSSION

In this study, we evaluated the effects of HIT and IRT in a group of active elderly men on cardiovascular fitness, on the main risk factors associated with metabolic syndrome and on muscle volume and strength.

HIT elicited significant modifications of selected anthropometrical features that carried over until the end of the resistance training. Similarly, several physiological parameters positively related with cardiovascular fitness and exercise capacity were improved after HIT and maintained after four months of recovery and after IRT. In addition, HIT elicited a significant decrease of systolic pressure, which was maintained also after strength training. Neither HIT nor IRT were able to induce relevant changes in blood lipid profile, with the exception of a decrease of LDL and CHO at the end of IRT. Moreover, both types of training were apparently sufficient to induce a substantial hypertrophy of the quadriceps muscle, which, however, was clearly paralleled by significant and increase in strength predominantly only after IRT. Therefore, some physiological parameters related with aerobic fitness and some selected body composition values predicting cardiovascular risk maintained at the end of detraining period the same values assessed after HIT.

The general message is that the improvements of physical-cardiovascular fitness, exercise capacity and of selected health related parameters obtained after HIT seem to be maintained after four months of recovery. After IRT, they may even be complemented by substantial increase of muscle and strength, leading to further improvements of the independency and quality of life of the subjects.

The following paragraphs are therefore devoted to critically discuss the observed findings and to outline some clinical and practical perspectives deriving from them.

### Anthropometry and body composition

8 weeks of HIT seems to be sufficient to induce a significant decrease of total body fat (BF %), android fat (AF %) and of W/height ratio (Table 1). These data are consistent with those presented by Stevensvold [41] who demonstrated the efficacy of 12 weeks of HIT in decreasing fat mass and WC in patients with metabolic syndrome. Moreover, they suggest that anthropometrical variables related to the risk of cardiovascular diseases or metabolic syndrome [42, 43] may be effectively abated also in healthy, active elderly subjects by HIT. After IRT, body composition remained basically the same as after HIT, confirming that resistance training can help maintaining the changes in regional body composition induced by aerobic training [44, 45].

Quadriceps CSA and Vol increased (4.3% ± 3.6 and 5.8% ± 4.6, respectively) after HIT. These findings are consistent with recent results [46] that showed a significant increase (+ 6%) of the quadriceps muscle volume in older men after 12 weeks of moderate- to-vigorous aerobic training paralleled by the increase of CSA of MHCI fibres. Whole muscle modifications were accompanied by beneficial myofiber modifications (specific MHCI peak force) that were more prominent in older subjects than in young subjects [46]. They also confirm the findings of a higher muscular thickness of vastus lateralis and intermedius subsequent to endurance training older adults [44]. Therefore, HIT may associate the beneficial effects on the cardiovascular system with those occurring at muscle level. In addition, the increased skeletal muscle volume would induce a high metabolic rate, decrease the accumulation of abdominal fat and lead to a higher disposal rate of glucose from the blood, thus decreasing the risk of insulin resistance and fasting hyperglycaemia.

Resistance training mainly led to significant increases of CSA and Vol (4.5% ± 4.4 and 5.3% ± 7.0) similar to those found after HIT. This confirms the results obtained in untrained elderly subjects in other occasions [42, 43].

Muscle morphological features, however, returned to baseline at the end of the recovery period. Therefore, it seems that the training stimuli at which the subjects were exposed during their active life between the two interventions were not sufficient to prevent the decline of muscle mass.

### Maximal oxygen uptake, anaerobic threshold and gross efficiency

The present study demonstrates that 8 weeks of HIT, applied three times a week in active, healthy elderly subjects with relatively brief bouts of exercise for a total of 14 minutes of intense exercise per session, is associated with significant changes of ${\dot{\text{V}}\text{O}}_{2\max}$ (9% ± 8) (Table 2). Several studies have already confirmed the efficacy of HIT in increasing ${\dot{\text{V}}\text{O}}_{2\max}$ in different populations [15, 50] and our results confirm the findings that 9 to 12 weeks of interval training can induce a significant increase of ${\dot{\text{V}}\text{O}}_{2\max}$ in sedentary aged subjects [47] and in patients with metabolic syndrome [41].

A low ${\dot{\text{V}}\text{O}}_{2\max}$ is a strong predictor of all-cause cardiovascular mortality [28] and each MET (Metabolic Equivalent Task) increase of ${\dot{\text{V}}\text{O}}_{2\max}$ may lead to a 8–17% drop of the mortality risk [48, 49]. Therefore, considering the time sparing characteristic of HIT and its benefit on ${\dot{\text{V}}\text{O}}_{2\max}$, this training modality might have a strong and positive impact on the quality of life and health of the older adults who show adaptations to aerobic training similar to those of young people [50].

The increase of ${\dot{\text{V}}\text{O}}_{2\max}$ after HIT was likely associated with the increase of ${\dot{\text{Q}}}_{\text{a}}{\text{O}}_{2\max}$, and with the amplification of maximal oxygen artero-veous difference, as suggested by the larger PO_2max_. However we know that older subjects underwent greater central modifications than young subjects during aerobic training [51]. Therefore, we may speculate that the increase of maximal cardiac output was the main central mechanism underpinning the observed increase of ${\dot{\text{V}}\text{O}}_{2\max}$. These results are consistent with those showing an increase of left ventricular mass and stroke volume in young adults [52] after high intensity training. ${\dot{\text{V}}\text{O}}_{2\max}$ per unit of body mass.

In agreement with other studies [53, 54], our data also show a non-significant decrease of ${\dot{\text{V}}\text{O}}_{2\max}$ after 16 weeks of detraining. However, they are in contrast with those of other investigators who found a rapid decay of relative ${\dot{\text{V}}\text{O}}_{2\max}$ after the interruption of aerobic training [55]. This may suggests that the main determinants of maximal aerobic fitness may be somehow resilient to the effects of detraining provided the subjects maintain an active life style.

HIT induced a significant improvement of the ventilatory threshold, specifically of RCP (19% ± 12%), which is highly correlated with to the critical power (CP) [56] that demarcates the heavy from severe intensity exercise domain. The improvement of RCP is correlated with the increase of performance time [57]. Our findings confirm those of previous investigations that showed the positive effects of HIT on the ventilatory threshold in elderly subjects [31, 58]. Several biochemical modifications may be advocated as the possible causes of the observed improvement in RCP. Indeed, two weeks of HIT have been shown to improve muscle oxidative capacity in humans as reflected by the increase of citrate synthase and cytochrome *c*-oxidase subunit IV [59] and by increasing the maximal activity and/or protein content of mitochondrial enzymes [60]. Also RCP did not diminish to attain, before resistance training, the level present before HIT confirming that elderly, but active, people seem to be somehow resilient to the detrimental effect of detraining on selected parameters related with aerobic fitness and exercise capacity.

IRT did not negatively affected ${\dot{\text{V}}\text{O}}_{2\max}$ and RCP, as these two parameters remained significant larger than the corresponding values assessed at Pre HIT (Table 2). These results are consistent with the findings of Frontera [17], who showed that 12 weeks of strength training elicited a significant improvement of ${\dot{\text{V}}\text{O}}_{2\max}$ in volunteers 60 to 72 years old in addition to the increase of muscular torque, As in that case training was followed by a 15% increase in capillary density and by a huge augmentation of citrate synthase activity (about + 40%), it seems that resistance training in elderly people may induce positive adaptations of the morphological and biochemical factors dictating peripheral gas exchanges and O_2_ utilization to such an extent to improve ${\dot{\text{V}}\text{O}}_{2\max}$ and exercise capacity.

### Blood analysis

Fasting GLU in all the subjects were below the threshold of 110 mg dl^−1^ (6.1 mmol L^−1^) and, therefore they were not affected by impaired glucose tolerance [61]. Nevertheless, 8 weeks of HIT were sufficient to induce a significant decay of GLU in our healthy elderly subjects. Similar improvements have been recently reported both after HIT training and continuous moderate aerobic exercise training in a wide spectrum of populations: healthy or overweight adults, overweight and obese adolescents, patients with metabolic syndrome [62, 63]. Since healthy older people may have an increased risk to develop Type 2 diabetes, HIT might postpone or avoid the occurrence of the disease in this population. Several mechanisms may responsible for the increase in insulin sensitivity after training. Among them, the increased abundance of type four glucose transporter (GLUT4) and the augmentation of insulin signalling in the muscle are nowadays considered the most important factors in improving insulin sensitivity [64]. In the present study, the decrease of GLU (%) induced by HIT was correlated with the increase (%) in LBM, suggesting that the increase of total muscle mass may help for a larger disposal of glucose even in a group of healthy and active elderly subjects. After, IRT, which was accompanied by a gain in LBM and by muscular hypertrophy (Table 1), GLU remained well below the values considered to diagnose stage I of impaired glucose tolerance. This would also confirm that resistance training has no specific contraindications as for the control of fasting glucose concentration. Yet, recent reviews and meta-analysis would confirm the efficacy of resistance training in abating and control GLU in Type II diabetes [65, 66].

Both HIT and IRT training intervention failed to produce relevant changes in blood lipid profile with the exception of LDL and CHO at the end of IRT. Although several studies analysed the effects of HIT on serum lipid profile [28], not many reported net positive outcomes on HDL cholesterol. For example 16 weeks of aerobic interval training (running) performed three times a week led to a relevant increase (18%) of HDL-C [66] in adults with metabolic syndrome. Conversely, a shorter period of 12 weeks of aerobic interval training did not bring about any significant change of the lipid profile, including HDL, in similar patients [41]. It has been proposed that the magnitude of the changes observed in HDL and its ratio to total cholesterol could be affected both by baseline values and by the duration of the phases of high intensity aerobic exercise [67]. Resistance training has been reported to modify lipid profile lowering CHO and LDL in middle-aged men [68], elderly subjects [69] and in Type II diabetes patients [70]. Therefore, even though it has been suggested that lipid profile benefits more from nutritional interventions than from exercise training [28], physical training (aerobic-resistance) may substantially co-operate with the effects of diet.

CRP concentration at the beginning of the training was, on the average, about 50% larger than that found in a group of men of similar age that included healthy subjects and patients with several prevalent diseases [71]. In addition, it was unaffected by HIT and, at the end of IRT it was significantly larger than the value at the baseline (Table 3). Only longer periods of concurrent aerobic and resistance exercise training [72] seem to be necessary to induce significant drops of CRP in elderly subjects [73]. The high value of CRP found after IRT might be the consequence of the muscle damage induced by the high load, concentric-eccentric resistance training [73]. Although we did not asses any biochemical marker of muscle damage, the per-cent increase of the count of leucocytes assessed at the very end of IRT was significantly correlated with the increase (%) of CRP (*r* = 0.62, *P* = 0.033, *n* = 12) indicating that an acute inflammatory condition was established, as it is the case when muscle damage occurs [74].

### Blood pressure

**Figure 1:**
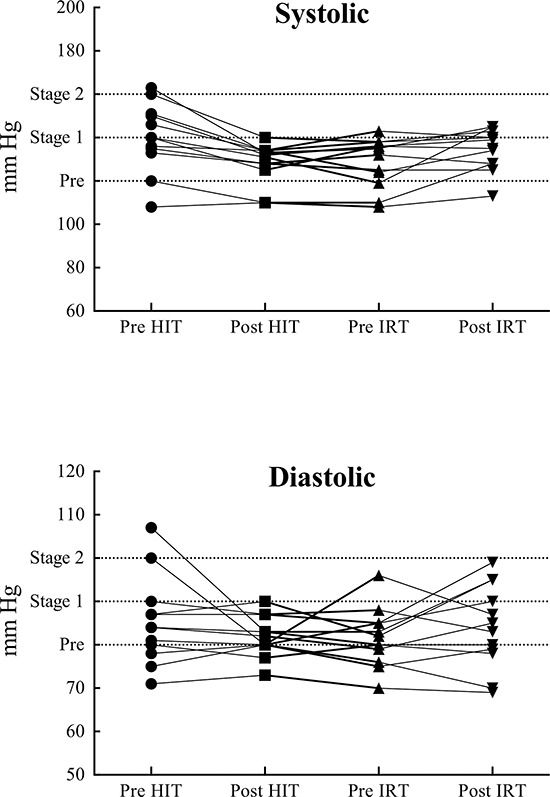
Systolic (upper panel) and diastolic (lower panel) blood pressure values at rest in the four evaluated conditions in the twelve subjects who participated in the study Dotted lines, from bottom to top, demarcate the passage from normal to prehypertension, from prehypertension to Stage 1 hypertension and from stage 1 to stage 2 hypertension.

At the beginning of the study, only one subject had normal blood pressure values according to the categories proposed by the American and International Societies of Hypertension (ASH/ISH) [75]. The lowering of the SYS observed in this study is consistent with previous results obtained after 12 weeks of HIT [76] or after traditional low intensity, large volume aerobic training in elderly men [77], but they are in contrast with the ones that did not demonstrate any decrease of SYS after HIT [41]. DP showed only marginal changes throughout the study, and MP was mainly affected by the consensual modifications of SYS. However, only SYS and DP are clinical meaningful for the diagnosis of prehypertension or hypertension of Stage 1 or 2 [75]. In this regard, before HIT 50% of patients satisfied the criteria for prehypertension, 8.3% for hypertension of stage 1 and 33.3% for hypertension of stage 2. After HIT, 75% of subjects belonged to the prehypertension categories, whereas normal and stage 1-hypertension groups were populated by one subject each. After recovery, the situation was only slightly modified: 58.3% of subjects were classified in the prehypertension, 16.7% in the normal and 25.0% in the stage 1-hypertension category. After resistance training, there was a substantial increase of the patients in the number of patients in the stage 1-hypertension class (58.3%), with a decrease of the ones present in the normal (8.3%) and prehypertension categories (33.3%) (Figure 1). These results confirm that dynamic resistance training seems not to adversely affect blood pressure in elderly trained subjects [77].

The decrease in SYS at rest and during submaximal exercise implies a decrease of the mechanical work of the heart and of the double product (SYS times HR) with a lower myocardial O_2_ demand. Meta analysis and observational studies have provided evidence that a blood pressure decrease of the same magnitude as that observed in this study are associated with a lower risk of vascular mortality (stroke or ischemic heart disease) [78].

### Muscular strength

IRT elicited, as reasonably expected, a significant and remarkable increase of maximal voluntary isometric torque and of the isokinetic concentric-eccentric torque of limb extensors. In general, muscular strength increased relative to the values prevailing at the beginning of the study and to the ones found before IRT. The increased in maximal voluntary torque was strongly-to-moderately correlated with the corresponding augmentation of Vol and CSA occurring over IRT, a finding suggesting the increase in strength was predominantly due to hypertrophy of the trained muscles. Resistance training has been found to be highly effective in increasing muscle strength both in older healthy men [79] and in patients with metabolic syndrome [41]. As the increase of strength was not accompanied by any substantial decrement or worsening of aerobic fitness, body size and composition and blood profile, we can also suggest that an intense strength training period subsequent to a HIT intervention does not seem to abate or nullify the beneficial effects of aerobic training in healthy older men. Rather, by increasing strength of postural and loco-motor muscles, it may further improve their quality of live and independency.

## MATERIALS AND METHODS

### Subjects

Twelve moderately active white, elderly male volunteers (age: 65–75 years) were recruited by local advertisements in the metropolitan area of Verona (Italy). Exclusion criteria were abnormal ECG at rest and during exercise, uncontrolled hypertension, diagnosis of cardiovascular, respiratory and metabolic diseases (recent heart infarction, unstable angina, uncompensated heart failure, severe valvular illness, diabetes, cardiomyopathy, asthma, COPD), moderate-severe renal failure, neurological and orthopaedic diseases limiting mobility and exercise, anti-coagulants and anti-aggregant therapy, alcohol and drug abuse and common contraindications to Magnetic Resonance Imaging (i.e. pacemakers, metallic clip). All of the subjects underwent a preliminary medical examination, to evaluate possible exclusion criteria and a preliminary cycle-ergometer stress test, to exclude pathological responses to exercise.

### Ethics statement

Investigation has been conducted in accordance with the ethical standards, with the Declaration of Helsinki, according to national and international guidelines and has been approved by the authors' institutional review board (approval on June 18^th^ 2013). Informed consent has been obtained.

### Experimental design

The study was a Single-Factor Within-Subjects design. All the subjects were evaluated twice before the HIT and IRT trainings for baseline values and immediately after the eight weeks of HIT (Post HIT) and IRT (Post IRT). Before the first data collection, all the subjects underwent a familiarization session where all the experimental procedures were thoroughly explained and carried out in a dry run, brief test session. Between HIT and IRT interventions, the subjects resumed for four months their normal daily activity habits (detraining).

The repeated control sessions allowed us to take into proper consideration the possible effect of the regression to the mean phenomenon, especially for the maximal exercise parameters, and their averages were considered as the control values of the two training interventions. Body composition and Magnetic Resonance (MRI) scans were evaluated only once before HIT and IRT and after training.

The tests in each experimental session were carried out in four subsequent days in the morning. In the first day, blood sampling for ematochemical assays and the main anthropometrical measurements were carried out. ${\dot{\text{V}}\text{O}}_{2\max}$ was then measured in the second day. In the third day, the volunteers performed six minutes of submaximal cycling exercise for the assessment of the gross mechanical efficiency of cycling. The last day was devoted to DXA measurements, MRI scans of the muscles of the thighs and to the 3-D total body scans.

### Training protocols

#### High Interval Training (HIT)

Volunteers trained three times a weeks for eight weeks. Training consisted in seven 2-minute bouts of cycling exercise (915 E, Monark, Varberg, S) at about 85–95% of individual ${\dot{\text{V}}\text{O}}_{2\max}$ interspersed by recovery intervals of identical duration at about 40% of ${\dot{\text{V}}\text{O}}_{2\max}$. This type of HIT, characterized by relatively long exercise bouts, performed at a high yet submaximal intensity, is commonly referred to as aerobic interval training (AIT) [28]. Each series was preceded by 10 minutes of active stretching and warm up. The entire training session lasted from 45 to 50 minutes, including the post training subjects cooling-down phase.

#### Isoinertial resistance training (IRT)

Bilateral resistance exercise was performed with the use of leg press flywheel ergometer [33] (YoYo Technology AB, Stockholm, Sweden) three times a week for 8 weeks. For the purpose of training, a configuration constructed for the seated knee extension was employed. Each session consisted of four sets of seven maximal, coupled concentric extensions and eccentric flexions of the knee from about 90° to 160°–170° knee joint angle. Sets, interspersed by 3-min rest periods, were initiated immediately following two submaximal actions. Any exercise session was preceded by a 10-min warm-up including three sets of seven submaximal actions with progressively increased effort. Training was performed using one polymer flywheel (4.2 kg). Any exercise session, including warm-up and rest periods, was completed in about 15 min.

### Anthropometry and body composition

Body mass and stature were taken at the nearest 0.1 kg and 0.01 m, respectively, with a Tanita electronic scale BWB-800 MA (Wunder SA.BI. Srl) and a stadiometer (Holtain Ltd., Crymych, Pembs. UK). Body mass index (BMI, kg m^−2^) was calculated as body mass/stature^2^. Waist circumference (minimum circumference of the waist, WC) was measured by 3D human scans based on structured light (Breuckmann Bodyscan^®^) using dedicated software on a VTK library. Total body was evaluated by means of dual-energy X-ray absorptiometry (DXA) using a total body scanner (QDR Explorer W, Hologic, MA, USA; fan-bean technology, software for Windows XP version 12.6.1) according to the manufacturer's procedures. The scanner was calibrated daily against the standard supplied by the manufacturer to avoid possible baseline drift. No special preparation was required, with the exception that participants had to wear underwear and not wear any metal accessories. The effective radiation doses involved are small (15.5 cGY cm^−2^), making the technique widely applicable.

Whole body scanning time was about seven minutes. Scanning and analyses were performed in the morning by the same operator (CM) in order to ensure consistency.

### Maximal oxygen uptake and anaerobic threshold

${\dot{\text{V}}\text{O}}_{2\max}$ and ventilatory thresholds were evaluated during a ramp test performed on an electronically braked cycle ergometer (Excalibur Sport, Lode, Groningen, The Netherlands). The incremental test consisted in 3 min at rest and 3 min of warm-up at 30 W followed by a continuous increment, every 1 min, of the workload by 10–15 W, depending on the prospective training status of each subject, until voluntary exhaustion. The latter was defined as the inability to maintain the pedalling frequency (60–80 revolutions/min), despite vigorous encouragement by the experimenters. Workload continuous increment was set a priori so that the subjects achieved voluntary exhaustion in 10–12 minutes. Then, after five minutes of recovery at 30 W, the subject started pedalling again a constant-work rate until exhaustion equal to 105% of the mechanical power achieved at the end of the ramp test [34]. This procedure allowed us to assess the individual ${\dot{\text{V}}\text{O}}_{2\max}$ without the need of secondary validating criteria.

The ergometer was operated by a metabolic cart (Quark b^2^, Cosmed, Rome, Italy) that allowed also continuous, breath-by-breath measures of gas exchange (at the mouth), ventilation and HR. Before each test, the gas analyzers and the turbine flow meter of the system were calibrated, following the manufacturer's instructions, by using a gas mixture of known concentration (FO_2_: 0.16; FCO_2_: 0.05; N_2_ as balance) and a 3.0-litre calibrated syringe.

${\dot{\text{V}}\text{O}}_{2\max}$ was assessed as the mean of the O_2_ values occurring in the last 30 seconds of the constant-work-rate test before the interruption. Gas exchange threshold (GET) and respiratory compensation point (RCP) were determined from the analysis of the gas exchanges [35] after a preliminary smoothing obtained by applying a three-sample moving average. Maximal oxygen pulse (PO_2_) was calculated as the ratio between ${\dot{\text{V}}\text{O}}_{2\max}$ and HR_max_, as obtained in the same temporal window during maximal test.

### Exercise efficiency

Gross exercise efficiency was calculated, by applying standard calorimetry, as the ratio between mechanical workload and metabolic power measured at steady state during 6 minutes of constant workload cycling exercise. Mechanical power was set to elicit a ${\dot{\text{V}}\text{O}}_{2}$ equal to the 80% of the first ventilatory threshold. Steady state ${\dot{\text{V}}\text{O}}_{2}$ was measured as the average of the breath-by-breath values during the last minute of exercise and converted in watt taking in proper account the caloric equivalent of O_2_ (CalO_2_) as: CalO_2_ = 4.686 + [(RER − 0.707) / 0.293], where RER represents the respiratory exchange ratio at steady state.

### Blood analysis

Blood samples were taken in the morning, in a fasting state. Blood was collected into siliconized vacuum tubes containing either K2 EDTA (Becton-Dickinson, Oxford, UK) for blood count analysis (Advia 2120, Siemens, Germany) or lithium heparin for clinical chemistry testing (Modular Analytics, Roche, CH). Clinical chemistry and immunochemistry tests were performed on Cobas^®^ 6000 c501 and e601 module (Roche Diagnostics GmbH, Penzberg, Germany), according to the manufacturer's specifications and using proprietary reagents. The panel of tests included the following: fasting glucose concentration (GLU), total cholesterol (CHOL), HDL cholesterol (HDL), LDL cholesterol (LDL), triglycerides (TRY). In addition, also c-reactive protein (CRP) and haemoglobyn (Hb) were obtained by standard methods. The instrument was calibrated against appropriate proprietary reference standard materials and verified with the use of proprietary quality controls. Our evaluation of the within-run precision by internal quality control on the Cobas^®^ 6000 c501 and e601module (Roche Diagnostics GmbH) showed low coefficients of variation.

### Blood pressure

Systolic (SYS) and diastolic (DP) blood pressures at rest in sitting position was measured before maximal test in the brachial artery by means of a blood pressure monitor (Tango+, SunTech Medical, Morrisville, NC, USA). Mean pressure was calculated as 1/3 (SYS-DP) + DP.

### MRI scan

After 1 h of supine rest to control for the influence of posturally related fluid shifts on muscle size, MRI scans were obtained for each subject. Subjects were supine, and their heels were fixed on a non-metallic support to control joint and scan angle and to minimize compression of the legs against each other and the MRI gurney. Imaging was completed in a 1.5 Tesla Magnetom Symphony (Siemens, Erlangen, Germany) to determine the volume (Vol) and cross-sectional area (CSA) of the total quadriceps *femoris*, *rectus femoris* (RF), *vastus lateralis* (VL), *vastus intermedius* (VI), and *vastus medialis* (VM) of the dominant limb. A coronal scout scan [repetition time/echo time (TR/TE) 5 300/14 ms, field of view 48 cm, 256 × 160 matrix] of 5 slices of 5 cm thick with 5-mm spacing was completed to establish orientation of the femur. After the scout scan, interleaved transaxial images of 1 cm thick (TR/TE 633/20 ms, field of view 274 × 480 mm, 256 × 256 matrix) were taken from the top of the greater trochanter of the femur to the articular surface of the tibia.

Magnetic resonance images were transferred electronically from the scanner to a personal computer (Macintosh Power PC) and analyzed with OsiriX (version 3.7.1 32 bit) using manual planimetry. Analyses of the magnetic resonance images began with the first proximal slice not containing gluteal muscle and continued distally to the last slice containing RF [36], because this region has been shown to represent the maximal CSA of the thigh [38]. The dominant leg of each subject was analyzed for CSA. The CSA of each of the four heads of the quadriceps femoris was manually outlined on each slice of thigh corresponding to the midpoint between the greater trochanter and the superior pole of the patella [38]. The average CSA (cm^2^) was taken as the average of all the analyzed slices for an individual muscle and determined for the RF, VL, VI, and VM and summed to obtain total Vol of the quadriceps femoris [39]. The same investigator carried out all the measurements. The reliability of this measurement has been assessed over 5 separate measurements of the cross-sectional area (CSA) of three heads of the quadriceps muscle taken distally at 50% of the femur bone length; the average coefficient of variation measuring the same image was 0.92% for the total quadriceps femoris.

### Statistics

Data are shown as means with SDs. Differences between conditions were evaluated by using one way analysis of variance for repeated measurements in a single within-subject factor design (GraphPad Prism version 6.00, CA, USA). Conservative F test was always calculated applying the Geisser and Greenhouse correction in order to take into consideration the condition of the sphericity of the data. Effect size was estimated by calculating partial omega squared (${\hat{\omega }}_{\varphi }^{2}$), which expresses the ratio between explained variability to total variability in terms of the population characteristics [40]. In addition, tests of specific differences based on the analysis of within-subject contrasts (*φ*) were carried out by using an Excel spread sheet programmed according to the algorithms indicated by Keppel and Wickens, including the contrast partial omega squared [40]. In particular, the considered contrasts were: Pre HIT vs. Post HIT, Pre IRT vs Post IRT, Pre HIT vs. Pre IRT, Post HIT vs. Post IRT. Correlation between variables was evaluated by means of the Perason's correlation coefficient. *P* value was always set at 0.05.

## CONCLUSIONS

Although not freed from methodological weaknesses, this longitudinal study may bring some new insightful indications and practical guidelines regarding the correlations between the effects induced by high intensity aerobic and resistance training.

Indeed, the experimental design (a single-factor, within-subjects design) prevented us to analyse the possible interaction between the effects induced by the two interventions. Moreover, the study was not counterbalanced. As such, he suffered from intrinsic limitations, since the results were not freed from incidental effects other than the one directly induced by the interventions. Finally, seasonal effects induced by the change of daily life activity may have acted as additional nuisance factors.

**Figure 2:**
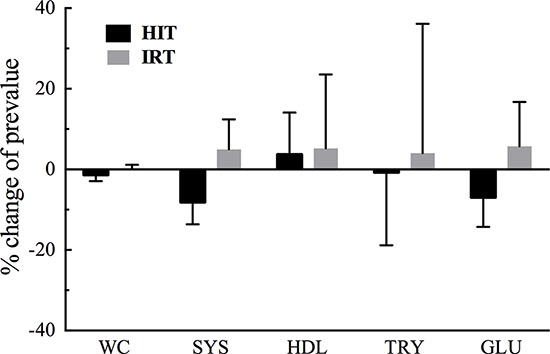
Mean per cent changes of the five risk factors defining metabolic syndrome induced by HIT and IRT in our subjects

Nevertheless, the results showed that HIT is effective in improving cardiovascular fitness and exercise capacity and to induce beneficial changes of several of the main five risk factors defining metabolic syndrome (Figure 2). Furthermore, provided the subjects are able to maintain an active life-style, we confirmed that the factors affecting cardiovascular fitness may not be adversely affected by long periods of detraining. Finally, resistance training induced a hypertrophic response of quadriceps that led to the consequent significant increase of maximal isometric and dynamic strength. The hypertrophic and functional adaptations of the trained muscle further contributed to improve the general fitness of the subjects and did not substantially interfere with the effect of HIT, since many of the changes induced by this sort of aerobic training were largely preserved.

## References

[1] Centers for Disease Control and Prevention (CDC) (2010). National Health and Nutrition Examination Survey Data 2003–2004, 2005–2006.

[2] Scholze J, Alegria E, Ferri C, Langham S, Stevens W, Stevens W, Jeffries D, Uhl-Hochgaeber K (2010). Epidemiological and economical burden of metabolic syndrome and its consequences in patients with hypertension in Germany, Spain and Italy; a prevalence-based model. BMC Public Health.

[3] Regitz-Zagrosek V, Lehmkuhl E, Weickert MO (2006). Gender differences in the metabolic syndrome and their role for cardiovascular disease. Clin Res Cardiol.

[4] Hamburg NM, Larson MG, Vita JA, Vasan RS, Keyes MJ, Widlansky ME, Fox CS, Mitchell GF, Levy D, Meigs JB, Benjamin EJ (2008). Metabolic syndrome, insulin resistance, and brachial artery vasodilator function in Framingham Offspring participants without clinical evidence of cardiovascular disease. Am J Cardiol.

[5] National Cholesterol Education Program (2002). Third report of the National Cholesterol Education Program (NCEP) Expert Panel on Detection, Evaluation, and Treatment of High Blood Cholesterol in Adults (Adult Treatment Panel III) final report. Circulation.

[6] Finley CE, LaMonte MJ, Waslien CI, Barlow CE, Blair SN, Nichaman MZ (2006). Cardiorespiratory fitness, macronutrient intake, and the metabolic syndrome: the Aerobics Center Longitudinal Study. J Am Diet Assoc.

[7] Isomaa B, Almgren P, Tuomi T, Forsen B, Lahti K, Nissén M, Taskinen MR, Groop L (2001). Cardiovascular morbidity and mortality associated with the metabolic syndrome. Diabetes Care.

[8] Lakka HM, Laaksonen DE, Lakka TA, Niskanen LK, Kumpusalo E, Tuomilehto J, Salonen JT (2002). The metabolic syndrome and total and cardiovascular disease mortality in middle-aged men. J Am Med Assoc.

[9] USDHHS (2008). Physical activity guidelines for Americans.

[10] Duscha BD, Slentz CA, Johnson JL, Houmard JA, Bensimhon DR, Knetzger KJ, Kraus WE (2005). Effects of exercise training amount and intensity on peak oxygen consumption in middle-age men and women at risk for cardiovascular disease. Chest.

[11] Myers J, Prakash M, Froelicher V, Do D, Partington S, Atwood JE (2002). Exercise capacity and mortality among men referred for exercise testing. N Engl J Med.

[12] Kusy K, Zielinsky J (2014). Aerobic capacity in speed-power athletes aged 20–90 years vs endurance runners and untrained participants. Scand J Med Sci Sports.

[13] Narici M, Maffulli N (2010). Sarcopenia: characteristics, mechanisms and functional significance. British Medical Bulletin.

[14] Fleg JL, Lakatta EG (1988). Role of muscle loss in the age associated reduction in ${\dot{\text{V}}\text{O}}_{2\max}$. J Appl Physiol.

[15] Balagopal P, Rooyackers OE, Adey DB, Ades PA, Nair KS (1997). Effects of aging on in vivo synthesis of skeletal muscle myosin heavy-chain and sarcoplasmic protein in humans. Am J Physiol.

[16] Fiatarone MA, Marks EC, Ryan ND, Meredith CN, Lipsitz LA, Evans WJ (1990). High-intensity strength training in nonagenarians. Effects on skeletal muscle. J Am Med Assoc.

[17] Frontera WR, Hughes VA, Lutz KJ (1991). A cross sectional study of muscle strength and mass in 45- to 78-yr-old men and women. J Appl Physiol.

[18] Jubrias SA, Oddeson IR, Esselman PC, Conley KE (1997). Decline in isokinetic force with age: muscle cross-sectional area and specific force. Pflugers Arch.

[19] Klitgaard H, Mantoni M, Schiaffino S, Ausoni S, Gorza L, Laurent-Winter C, Schnohr P, Saltin B (1990). Function, Morphology and protein expression of ageing skeletal muscle: a cross-sectional study of elderly men with different training back-grounds. Acta Physiol Scand.

[20] Vaughan L, Zurlo F, Ravussin E (1991). Aging and energy expenditure. Am J Clin Nutr.

[21] Goran MI, Poehlman ET (1992). Total energy expenditure and energy requirements in healthy elderly persons. Metabolism.

[22] Kohrt WM, Holloszy JO (1995). Loss of skeletal muscle mass with aging: effect on glucose tolerance. J Gerontol A Biol Sci Med Sci.

[23] di Prampero PE, Ferretti G (1990). Factors limiting maximal oxygen consumption in humans. Respir Physiol.

[24] Ferretti G, Capelli C (2009). Maximal O2 consumption: Effects of gravity withdrawal and resumption. Resp Physiol Neurobiol.

[25] Centers for Disease Control and Prevention (2010). State indicator report on physical activity 2010 Division of Nutrition, Physical Activity, and Obesity.

[26] McDonald P, Kippen R (2001). Labor supply prospects in 16 developed countries 2000–2050. Pop Developm Rev.

[27] Gibala MJ, Little JP, MacDonald MJ, Hawley JA (2012). Physiological adaptations to low-volume, high-intensity interval training in health and disease. J Physiol.

[28] Kessler HS, Sisson Sb, Short KR (2012). The potential for High Intensity Interval Training to reduce cardio metabolic disease risk. Sports Med.

[29] Pattyn N, Cornelissen VA, Eshghi SR, Vanhees L (2013). The effect of exercise on the cardiovascular risk factors constituting the metabolic syndrome: a meta-analysis of controlled trials. Sports Med.

[30] Cornelissen VA, Arnout J, Holvoet P, Fagard RH (2009). Influence of exercise at lower and higher intensity on blood pressure and cardiovascular risk factors at older age. J Hypertension.

[31] Ahmaidi S, Masse-Biron J, Adam B, Choquet D, Freville M, Libert JP, Prefaut C (1998). Effects of interval training at the ventilatory threshold on clinical and cardiorespiratory responses in elderly humans. Eur J Appl Physiol Occup Physiol.

[32] Tesch P, Ekberg A, Lindquist D, Trieschmann J (2004). Muscle hypertrophy following 5-week resistance training. Acta Physiol Scand.

[33] Berg H, Tesch A (1994). A gravity-independent ergometer to be used for resistance training in space. Aviat Space Environ Med.

[34] Poole DC, Wilkerson DP, Jones AM (2008). Validity of criteria for establishing maximal O2 uptake during ramp exercise test. Eur J Appl Physiol.

[35] Beaver WL, Wasserman K, Whipp BJ (1986). A new method for detecting anaerobic threshold by gas exchange. J Appl Physiol.

[36] Castro MJ, Apple DF, Hillegass EA, Dudley GA (1999). Influence of complete spinal cord injury on skeletal muscle cross sectional area within the first 6 mo of injury. Eur J Appl Physiol.

[37] Narici MV, Roi GS, Landoni L (1988). Force of knee extensor and flexor muscles and cross-sectional area determined by nuclear magnetic resonance imaging. Eur J Appl Physiol.

[38] Harridge S, Kryger A, Stensgaard A (1999). Knee extensor strength, activation, and size in very elderly people following strength training. Muscle Nerve.

[39] Trappe TA, Lindquist DM, Carrithers JA (2001). Muscle-specific atrophy of the quadriceps femoris with aging. J Appl Physiol.

[40] Keppel G, Wickens TD (2004). Design and Analysys. A researcher's handbook.

[41] Stevensvold D, Tjønna AE, Skaug EA, Aspenes S, Stølen T, Wisløff U, Slørdahl SA (2010). Strength training versus aerobic interval training to modify risk factors of metabolic syndrome. J Appl Physiol.

[42] Lee CMY, Huxley RR, Wildman RP, Woodward M (2008). Indices of abdominal obesity are better discriminators of cardiovascular risk factors than BMI: a meta-analysis. J Clin Epidem.

[43] Shunkert H, Moebus S, Hanisch J, Steinhagen-Thiessen E, Hauner H, Weil J, Wasem J, Jöckel KH (2008). The correlation between waist circumference and ESC cardiovascular risk score: data from the German metabolic and cardiovascular risk project (GEMCAS). Clin Res Cardiol.

[44] Sillanpää E, Häkkinen A, Nyman K, Mattila M, Cheng S, Karavirta L, Laaksonen DE, Huuka N, Kraemer WJ, Häkkinen K (2008). Body Composition and Fitness during Strength and/or Endurance Training in Older Men. Med Sci Sports Exerc.

[45] Treuth MS, Ryan AS, Pratley RE, Rubin MA, Miller JP, Nicklas BJ, Sorkin J, Harman SM, Goldberg AP, Hurley BF (1994). Effects of strength training on total and regional body composition in older men. J Appl Physiol.

[46] Harber MP, Konopka AR, Undem MK, Hinkley JM, Minchev K, Kaminsky LA, Trappe TA, Trappe S (2012). Aerobic exercise training induces skeletal muscle hypertrophy and age-dependent adaptations in myofiber function in young and older men. J Appl Physiol.

[47] Lepretre PM, Vogel T, Brechat PH, Dufour S, Richard R (2009). Impact of short-term aerobic interval training on maximal exercise in sedentary aged subjects. Int J Clin Pract.

[48] Myers J, Prakash M, Froelicher V, Do D, Partington S, Atwood JE (2002). Exercise capacity and mortality among men referred for exercise testing. N Engl J Med.

[49] Blair SN, Kohl HW, Barlow CE, Paffenbarger RS, Gibbons LW, Macera CA (1995). Changes in physical fitness and all-cause mortality. A prospective study of healthy and unhealthy men. J Am Med Assoc.

[50] Kohrt W, Malley MT, Coggan AR, Spina RJ, Ogawa T, Ehsani AA, Bourey RE, Martin WH, Holloszy JO (1991). Effects of gender, age, and fitness level on response of ${\dot{\text{V}}\text{O}}_{2\max}$ to training in 60–71 yr olds. J. Appl. Physiol.

[51] Murias JM, Kowalchuk JM, Paterson DH (2010). Time course and mechanisms of adaptations in cardiorespiratory fitness with endurance training in older and young men. J Appl Physiol.

[52] Matsuo T, Saotome K, Seno S, Shimojo N, Matsushita A, Iemitsu M, Ohshima H, Tanaka K, Mukai C (2013). Effects of a Low-Volume Aerobic-Type Interval Exercise on ${\dot{\text{V}}\text{O}}_{2\max}$ and Cardiac Mass. Med Sci Sports Exerc.

[53] Ratel S, Gryson C, Rance M, Penando S, Bonhomme C, Le Ruyet P, Duclos M, Boirie Y, Walrand S (2011). Detraining-induced alterations in metabolic and fitness markers after a multicomponent exercisetraining program in older men. Appl Physiol Nutr Me.

[54] Sforzo GA, McManis BG, Black D, Luniewski D, Scriber KC (1995). Resilience to exercise detraining in healthy older adults. J Am Geriatr Soc.

[55] Simoneau JA, Lortie G, Boulay MR, Marcotte M, Thibault MC, Bouchard C (1987). Effects of two high-intensity intermittent training programs interspaced by detraining on human skeletal muscle and performance. Eur J Appl Physiol.

[56] Bergstrom HC, Housh TJ, Zuniga JM, Traylor DA, Camic CL, Lewis RW, Schmidt RJ, Johnson GO (2013). The relationships among critical power determined from a 3-min all-out test, respiratory compensation point, gas exchange threshold, and ventilatory threshold. Res Q Exerc Sport.

[57] Black MI, Durant J, Jones AM, Vanhatalo A (2014). Critical power derived from a 3-min all-out test predicts 16.1-km road time-trial performance. Eur J Sport Sci.

[58] Pogliaghi S, Terziotti P, Cevese A, Balestreri F, Schena F (2006). Adaptations to endurance training in the healthy elderly: arm cranking versus leg cycling. Eur J Appl Physiol.

[59] Hood M, Lilltel JP, Tarnopolsky MA, Myslik F, Gibala MJ (2001). Low-Volume interval training improves muscle oxidative capacity in sedentary adults. Med Sci Sports Exerc.

[60] Burgomaster KA, Hughes SC, Heigenhauser GJ, Bradwell SN, Gibala MJ (2005). Six sessions of sprint interval training increases muscle oxidative potential and cycle endurance capacity in humans. J Appl Physiol.

[61] (1997). The Expert Committee on the Diagnosis and Classifications of Diabetes Mellitus. Diabetes Care.

[62] Pattyn N, Cornelissen VA, Eshghi SR, Vanhees L (2013). The effect of exercise on the cardiovascular risk factors constituting the metabolic syndrome: a meta-analysis of controlled trials. Sports Med.

[63] Wallman K, Plant LA, Rakimov B, Maiorana AJ (2009). The effects of two modes of exercise on aerobic fitness and fat mass in an overweight population. Res Sports Med.

[64] Pedersen BK (2009). The diseasome of physical inactivity – and the role of myokines in muscle – fat cross talk. J Physiol.

[65] Gordon BA, Benson AC, Bird SR, Fraser SF (2009). Resistance training improves metabolic health in type 2 diabetes: A systematic review. Diabetes res Clin Pract.

[66] Tjønna AE, Lee SJ, Rognmo O, Stølen TO, Bye A, Haram PM, Loennechen JP, Al-Share QY, Skogvoll E, Slørdahl SA, Kemi OJ, Najjar SM, Wisløff U (2008). Aerobic interval training versus continuous moderate exercise as a treatment for the metabolic syndrome: a pilot study. Circulation.

[67] Stampfer MJ, Sacks FM, Salvini S, Willett WC, Hennekens CH (1991). A prospective study of cholesterol, apolipoproteins, and the risk of myocardial infarction. N Engl J Med.

[68] Libardi A, Bonganha V, Soares Conceição M, Verginia De Souza V, Ferandes Bernardes C, Secolin R, Aparecida MAdruga V, Traina Chacon-Mikahil MP (2012). The periodized resistance training promotes similar changes in lipid profile in middle-aged men and women. J Sports Med Phys Fitnsess.

[69] Kim HS, Kim DG (2013). Effect of long-term resistance exercise on body composition, blood lipid factors, and vascular compliance in the hypertensive elderly men. J Exerc Rehab.

[70] Honkola A, Forsén T, Eriksson J (1997). Resistance training improves the metabolic profile in individuals with type 2 diabetes. Acta Diabet.

[71] Ahmadi-Abhari S, Luben RN, Wareham NJ, Khaw KT (2013). in the older adult population: European Prospective Investigation into Cancer-Norfolk study. Eur J Clin Invest.

[72] Stewart LK, Flynn MG, Campbell WW, Craig BA, Robinson JP, Timmerman KL, McFarlin BK, Coen PM, Talber E (2007). The Influence of Exercise Training on Inflammatory Cytokines and C-Reactive Protein. Med Sci Sports Exerc.

[73] Martins RA, Neves AP, Coelho-Silva MJ, Veríssimo MT, Teixeira AM (2010). The effect of aerobic versus strength-based training on high-sensitivity C-reactive protein in older adults. Eur J Appl Physiol.

[74] Mendham AE, Donges CE, Liberts EA, Duffield R (2011). Effects of mode and intensity on the acute exercise-induced IL-6 and CRP responses in a sedentary. Overweight population. Eur J Appl Physiol.

[75] Weber MA, Schiffrin EL, White WB, MANN S, Lindholm LH, Kenerson JG, Flack JM, Carter BL, Materson BJ, Ram CV, Cohen DL, Cadet JC, Jean-Charles RR, Taler S, Kountz D, Townsend R, Chalmers J, Ramirez AJ, Bakris GL, Wang J, Schutte AE, Bisognano JD, Touyz RM, Sica D, Harrap SB (2014). Clinical practice guidelines for the management of hypertension in the community: a statement by the American Society of Hypertension and the International Society of Hypertension. J Hypertension.

[76] Tjønna AM, Leinan IM, Thoresen Bartnes A, Jenssen BM, Gibala MJ, Winett RA, Wisløff U (2013). Low- and High-Volume of Intensive Endurance Training Significantly Improves Maximal Oxygen Uptake after 10-Weeks of Training in Healthy Men. PlosOne.

[77] Cononie CC, Graves CJE, Pollock ML, Philips MI, Sumners C, Hagberg JM (1991). Effect of exercise training on blood pressure in 70- to 79-yr-old men and women. Med Sci Sports Exerc.

[78] Lewington S, Clarke R, Qizilbash N, Peto R, Collins R (2002). Age-specific relevance of usual blood pressure to vascular mortality: a meta-analysis of individual data for one million adults in 61 prospective studies. Lancet.

[79] Hurley BF, Redmond RA, Pratley RE, Treuth MS, Rogers MA, Goldbeg AP (1994). Effects of Strength Training on Muscle Hypertrophy and Muscle Cell Disruption in Older Men. Int J Sports Med.

[80] Alison D, Hunter G, Lara-Castro C, St. Onge MP, Zakahrkin S (2005). Weight loss needed to maintain visceral adipose tissue during aging. Int. J. Body Comp. Res.

[81] Musa DI, Adeniran SA, Dikko AU, Sayers SP (2009). The effect of a high-intensity interval training program on high-density lipoprotein cholesterol in young men. J. Strength Cond. Res.

[82] Yang Z, Scott CA, Mao C, Tang J, Farmer AJ (2014). Resistance Exercise Versus Aerobic Exercise for Type 2 Diabetes: A Systematic Review and Meta-Analysis. Sports Med.

